# Epstein–Barr Virus and the Pathogenesis of Diffuse Large B-Cell Lymphoma

**DOI:** 10.3390/life13020521

**Published:** 2023-02-14

**Authors:** Aisling M. Ross, Ciara I. Leahy, Fiona Neylon, Jana Steigerova, Patrik Flodr, Martina Navratilova, Helena Urbankova, Katerina Vrzalikova, Lucia Mundo, Stefano Lazzi, Lorenzo Leoncini, Matthew Pugh, Paul G. Murray

**Affiliations:** 1 Health Research Institute and School of Medicine, University of Limerick, V94 T9PX Limerick, Ireland; 2 BioScience and BioEngineering Research (BioSciBer), Bernal BioMaterials Cluster, Bernal Institute, University of Limerick, V94 T9PX Limerick, Ireland; 3 Department of Clinical and Molecular Pathology, Institute of Molecular and Translational Medicine, Faculty of Medicine and Dentistry, Palacky University Olmouc, 775 15 Olomouc, Czech Republic; 4 Department of Clinical and Molecular Pathology, University Hospital Olomouc, 779 00 Olomouc, Czech Republic; 5 Department of Hemato-Oncology, Faculty of Medicine and Dentistry, Palacky Univesity and University Hospital Olomouc, 779 00 Olomouc, Czech Republic; 6 Institute of Immunology and Immunotherapy, University of Birmingham, Birmingham B15 2TT, UK; 7 Department of Medical Biotechnologies, Section of Pathology, University of Siena, 53100 Siena, Italy

**Keywords:** Epstein–Barr virus, diffuse large B-cell lymphoma, tumour microenvironment, chronic inflammation

## Abstract

Epstein–Barr virus (EBV), defined as a group I carcinogen by the World Health Organization (WHO), is present in the tumour cells of patients with different forms of B-cell lymphoma, including Burkitt lymphoma, Hodgkin lymphoma, post-transplant lymphoproliferative disorders, and, most recently, diffuse large B-cell lymphoma (DLBCL). Understanding how EBV contributes to the development of these different types of B-cell lymphoma has not only provided fundamental insights into the underlying mechanisms of viral oncogenesis, but has also highlighted potential new therapeutic opportunities. In this review, we describe the effects of EBV infection in normal B-cells and we address the germinal centre model of infection and how this can lead to lymphoma in some instances. We then explore the recent reclassification of EBV+ DLBCL as an established entity in the WHO fifth edition and ICC 2022 classifications, emphasising the unique nature of this entity. To that end, we also explore the unique genetic background of this entity and briefly discuss the potential role of the tumour microenvironment in lymphomagenesis and disease progression. Despite the recent progress in elucidating the mechanisms of this malignancy, much work remains to be done to improve patient stratification, treatment strategies, and outcomes.

## 1. Introduction

Diffuse Large B-cell Lymphoma (DLBCL) is the most prevalent subtype of non-Hodgkin lymphoma, accounting for approximately 25–35% of diagnosed cases [1,2,3]. In the last three decades, treatment strategies for DLBCL have improved through the addition of the anti-CD20 monoclonal antibody, rituximab, to the chemotherapy cocktail of cyclophosphamide, hydroxy-daunorubicin (doxorubicin), vincristine, and prednisone (R-CHOP), resulting in an increase in overall survival (OS) and progression-free survival (PFS). However, up to 40% of DLBCL patients have refractory or relapsed (R/R) disease which is eventually fatal in most cases [4,5,6]. 

**Figure 1:**
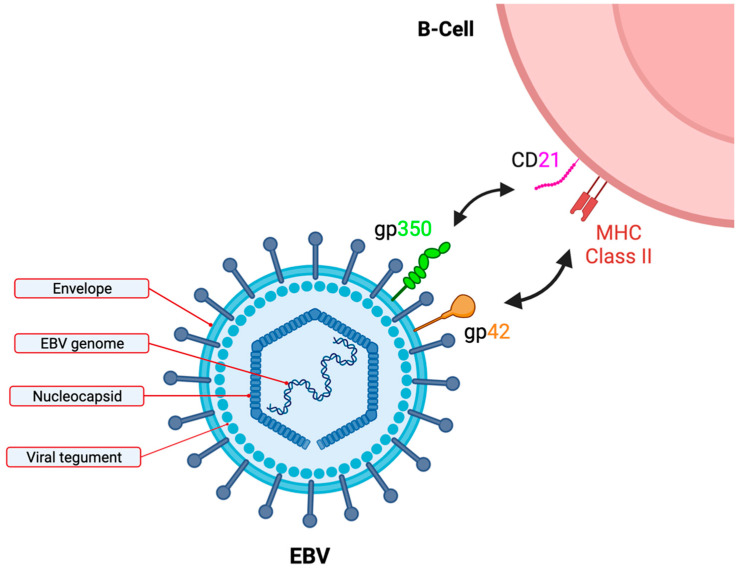
Schematic of B-cell infection by Epstein-Barr virus (EBV). EBV consists of linear, double-stranded DNA surrounded by a nucleocapsid layer, a protein tegument, and, finally, a viral envelope. The viral envelope includes the protein gp350 which binds to B-cells via the CD21 receptor on the B-cell surface, and the protein gp42 which binds to the MCH class II molecule. Created with BioRender.com.

A particularly poor prognosis subtype of DLBCL is those tumours that are associated with Epstein–Barr virus (EBV). EBV, formally named human herpesvirus 4 (HHV-4), is one of eight known human herpesviruses. As a herpesvirus, the genome of EBV is double-stranded, linear, and surrounded by a protein capsid. This is, in turn, surrounded by an envelope embedded with glycoproteins, and the intermediate space is filled with a protein tegument (Figure 1). The viral genome is approximately 172 kb in length and encodes over 85 genes [7,8,9]. Due to the oncogenic properties of a small subset of these 85 genes, EBV is associated with a number of human B-cell lymphomas, including Hodgkin lymphoma (HL), post-transplant lymphoproliferative disorder (PTLD), and, more recently, diffuse large B-cell lymphoma (DLBCL). Although the presence of EBV in DLBCL is relatively rare (approx. 5–15% of DLBCL tumours are diagnosed as EBV+), this malignancy is reportedly associated with poorer outcomes even after adjusting for confounding factors [10,11]. EBV+ DLBCL was first included in the World Health Organization (WHO) classification in 2008 as a provisional entity, under DLBCL, NOS, where it was restricted to a population >50 years of age (termed EBV+ DLBCL of the elderly) [12,13]. However, the age restriction was subsequently removed in 2016 and EBV+ DLBCL was reclassified as an established entity in 2022 [14]. 

The reclassification of EBV+ DLBCL as an established entity by WHO presents an opportunity to re-evaluate the evolution of EBV+ DLBCL classification over the last 15 years. Herein, we review the role of viral and cellular genetics on tumour development and explore possible roles of the tumour microenvironment in pathogenesis. Although progress has been made in improving our understanding of this malignancy, it is clear that further work is needed to better understand this disease if we hope to improve outcomes associated with this rare but aggressive malignancy.

## 2. EBV Is Transforming in B-Cells

EBV is an ancient virus which has co-evolved with humans for approximately 12 million years, proven by its similarity to Old World non-human primate lymphocryptoviruses (LCVs) [15]. Though the virus itself is ancient, it was only discovered in 1964 in a cell line (known as the EB1 cell line after Tony Epstein and Yvonne Barr) derived from a patient with Burkitt lymphoma (BL), an aggressive malignancy of germinal centre B-cells [16]. A critical question that followed this discovery was whether the virus contributed to the disease process or, as many suggested at the time, was merely an innocent bystander.

The uncertainty with which EBV was regarded as a new cancer-causing virus was fuelled by the results of the newly developed serological assays. These assays demonstrated that antibodies to EBV antigens were detectable in infectious mononucleosis (IM) sufferers [17] but also in most healthy individuals [18,19]. The frequency with which EBV was detected in healthy individuals, as well as its identification as the causative agent in IM, seemed incompatible with a potential role as a carcinogen. EBV virions are also found in the saliva of IM patients and at much lower levels in healthy seropositive people [20].

In early experiments to address the question of EBV’s transforming capabilities, it was shown that cord blood lymphocytes cultivated with irradiated EBV+ Jijoye BL cells, which produce virions, led to the growth of the lymphocytes [21]. It was also shown in the same year that immortalised B-cell lines, termed lymphoblastoid cell lines (LCL), could be established from the peripheral lymphocytes of EBV-infected individuals, confirming that EBV is a potent transformer of normal B-cells [22,23]. Further, it was established that tumour cells in EBV+ BL and EBV+ AIDS-related lymphoma contained clonal copies of EBV [24]. This indicated that the progenitor tumour cell carried EBV, thereby supporting that belief that EBV was involved in tumorigenesis [25]. This ultimately led to the designation of EBV as the first human virus linked to cancer [26,27].

**Figure 2:**
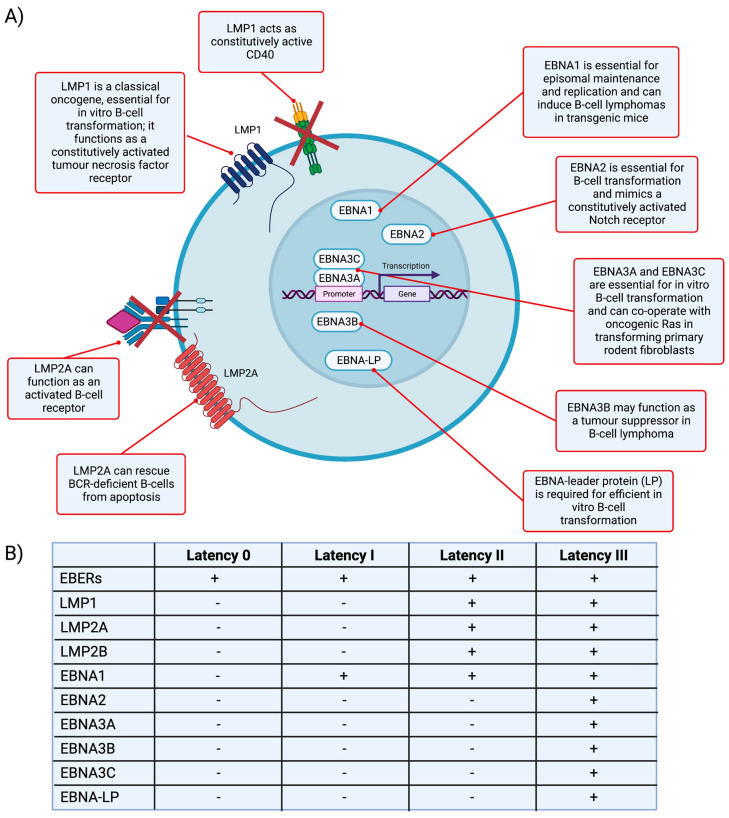
Major EBV latent proteins and their functions in B-cells. (**A**) The major EBV latent proteins, including the Epstein–Barr nuclear antigens (EBNAs) and the latent membrane proteins, LMP1 and LMP2A. LMP1 and LMP2A, in particular, act as constitutively active CD40 and B-cell receptor mimics, respectively, which are thought enable the cells to transition through the germinal centre reaction and exit as memory B-cells. The latent proteins are additionally important not only for EBV’s ability to transform healthy B-cells, but are also implicated in the pathogenesis of virus-associated B-cell malignancies, including diffuse large B-cell lymphoma. (**B**) Marker positivity for the different latency programs are shown in the table; “+” signifies expressed; “-” signifies not expressed. EBERs = Epstein–Barr virus-encoded RNAs (non-coding); LMP = latent membrane protein; EBNA = Epstein–Barr nuclear antigen; LP = leader protein; BCR = B-cell receptor. Created with BioRender.com.

Later, viral genomic sequencing studies began to elucidate the viral genes that were responsible for the in vitro transformation of B-cells [8]. We now know it is the coordinated expression of the so-called latent EBV genes that is responsible for EBV’s transforming potential. Established LCL express all latent genes (known as the ‘growth programme’ or latency III), which include six proteins located in the nucleus of infected cells (known as Epstein–Barr nuclear antigens; EBNAs 1, 2, 3A, 3B, 3C, LP), and three proteins present in the plasma membrane (known as latent membrane proteins (LMP1, LMP2A, and LMP2B) (Figure 2) [28,29]. In all infected cells, EBV also encodes the highly abundant non-coding Epstein–Barr virus-encoded RNAs (EBER1 and EBER2), which are used as a target in diagnostic assays to detect EBV [28,29]. EBV also encodes 44 viral miRNAs which can be clustered in two groups according to the region of the EBV genome from which they are derived: BART (40 miRNAs) and BHRF1 (4 miRNAs) [30,31,32]. These miRNAs are thought to contribute to oncogenesis, at least in part, by inhibiting translation of host mRNAs encoding tumour suppressors [31,33].

B-cells latently infected with EBV can also be induced into the lytic phase, which is accompanied by the shutting down of expression of most of the latent genes and the expression of a larger number of proteins that are required for the reproduction of the viral genome and the eventual construction and release of new virions.

### The Nature of Asymptomatic EBV Carriage in B-Cells

**Figure 3:**
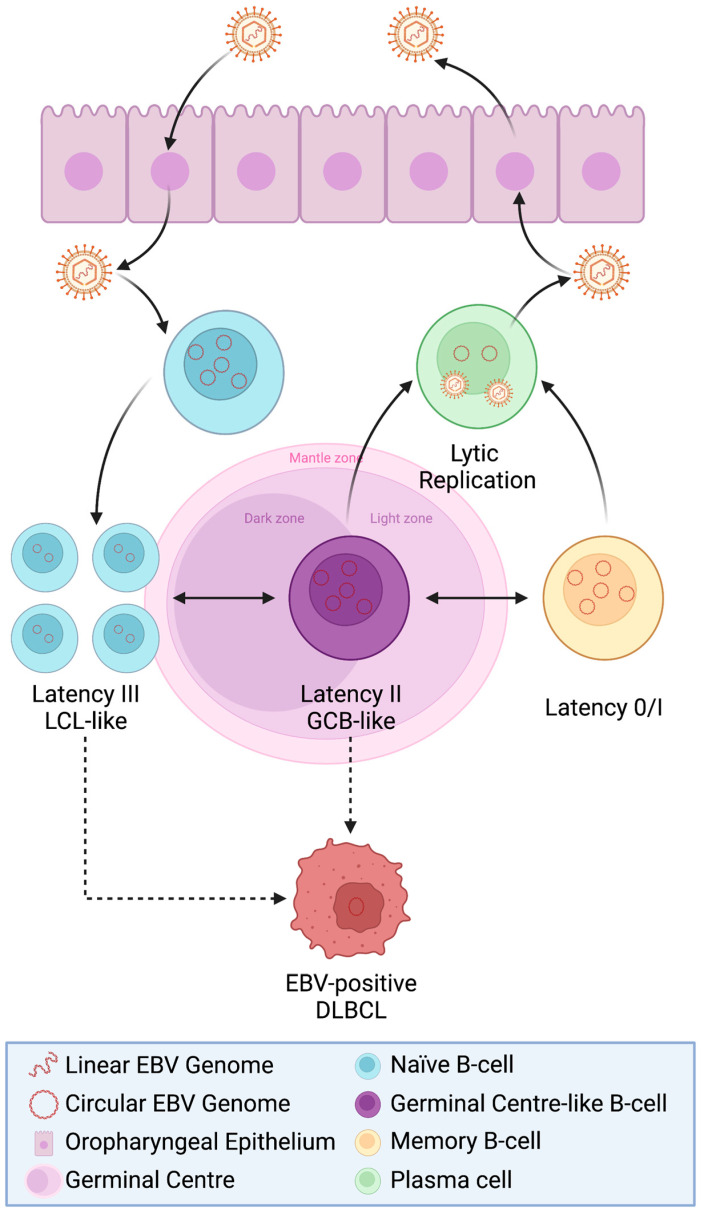
Origin of Epstein-Barr virus (EBV)-positive diffuse large B-cell lymphoma (DLBCL). Shown are the stages through which EBV-infected cells pass in the so-called germinal centre (GC) model of EBV persistence in the asymptomatic host and the associated latency programmes. LCL = Lymphoblastoid cell line. Created with BioRender.com.

The transmission of EBV is mainly via oral contact, but can also occur via intercourse [34,35,36]. To infect B-cells, EBV gp350 binds to the CD21 receptor (also known as CR2, complement C3d receptor, and the EBV receptor), widely expressed on the B-cell surface. The viral gp42 then interacts with the HLA class II molecules, which results in fusion with the host membrane (Figure 1) [37,38]. Infection can lead to IM or can be asymptomatic [9]. In asymptomatic carriers, memory B-cells have been shown to be the major cell type infected with EBV [39]. It has been suggested that memory B-cells may be directly infected with EBV [40,41] or that EBV initially infects naïve B-cells, which then differentiate to become memory B-cells. The latter provides an elegant model to explain EBV persistence (see [42,43] for a more detailed discussion of primary EBV infection and IM) and, in turn, also explains the distinct forms of latency which are tightly controlled in the different EBV-associated B-cell malignancies (Figure 3). In this model, EBV-infected naïve B-cells adopt a latency III programme that drives their proliferation. During the early stages of EBV infection, it is also thought that there may be expression of additional viral genes that promote cell survival, in addition to proliferation. In particular, Altmann and Hammerschmidt (2005) demonstrated that the expression of two viral homologs of BCL-2, a protein involved in pro-survival signalling, may be important for providing anti-apoptotic signals in newly infected B-cells [44]. They found that without these BCL-2 homologs, EBV-infected cells immediately underwent apoptosis, but that these proteins are no longer essential once latent infection is established [44].

Once infected, the B-cells differentiate in a germinal centre (GC)-like phase and express another form of latency, known as the ‘default programme’ or latency II, in which expression is limited to the LMPs and EBNA1 [45]. LMP1 and LMP2A would seem to be crucial here as they act as CD40 and B-cell receptor (BCR) mimics, respectively, ensuring that the EBV-infected cells can survive and exit the GC reaction, emerging as memory B-cells in which the EBV genome is maintained as an extra-chromosomal episome [32,46,47].

Thereafter, EBV-infected memory B-cells almost entirely shut down virus gene expression (known as latency 0) [45], but can switch on EBNA1 expression to allow the replication and segregation of viral episomes during the proliferation of memory B-cells (latency I). EBV-infected memory B-cells can differentiate into plasma cells and this is accompanied by induction of the virus lytic cycle [48].

In a normal host, adaptive T-cell responses effectively eliminate EBV-infected cells expressing viral antigens [27]. This appears to be especially important in latency III; in these cells, the immunodominant EBNA3 family of EBV proteins are expressed [27,49]. However, the loss of adaptive immunity, for example, in iatrogenically immunosuppressed individuals or in those with uncontrolled HIV infection, can lead to the outgrowth of latency III-expressing cells. This eventually leads to the development of latency III-type lymphomas [49]. Moreover, it is important to note that innate lymphocyte populations are also involved in the control of EBV in the asymptomatic host, as evidenced by the development of EBV-associated pathologies, including lymphomas, in people with primary immunodeficiencies associated with a loss of innate lymphocyte functions [50].

## 3. EBV+ Diffuse Large B-Cell Lymphoma

In the most recent lymphoma classifications, WHO fifth edition (WHO-HEAM5) and ICC 2022, a wide range of large B-cell lymphomas are recognised, showing a variable association with EBV (Table 1). The archetypal EBV-associated large B-cell lymphoma, EBV+ DLBCL, has long been regarded a separate entity from DLBCL, NOS, based on the unique clinical, morphological, and prognostic features. Defined as a large B-cell lymphoma in which the majority of the neoplastic cells harbour EBV without a prior history of immunodeficiency or dysregulation, EBV+ DLBCL was initially thought to mostly affect people over 50 years old (peak in seventh and eighth decades), and frequently presents with extranodal disease. The lung, gastrointestinal tract, skin, and bone marrow are the most frequently affected. Younger patients (peak in third decade) typically present with nodal disease. Histopathologically, EBV+ DLBCL shows a broad spectrum of features, ranging from monotonous sheets of large blasts to polymorphous infiltrates with variable-sized neoplastic B-cells and dense lymphoid infiltrates comprising lymphocytes, plasma cells, and epithelioid histiocytes. The lesional B-cells can even show Hodgkin-/Reed Sternberg-like features, leading to diagnostic confusion with Hodgkin lymphoma and other EBV+ lymphoproliferations (see [51] for a recent review of EBV-associated B-cell lymphoproliferative disorders). On immunophenotyping, the lesional cells express pan B-cell markers (CD20, CD19, PAX5) and approx. 40% of cases express CD30, which is significantly more than the EBV-negative cohort [52]. EBV+ tumoral status has been shown to be associated with more aggressive clinical behaviour in DLBCL patients; although, the prognostic effect may vary according to geographical region and within different age groups.

### 3.1. Evolution of DLBCL Classification and EBV

Before considering the role of EBV in the pathogenesis of EBV+ DLBCL, it is worth pausing briefly to consider the classification of large B-cell lymphomas, which has undergone, and continues to undergo, substantial modification. Lymphoma classification started in the first half of the 19th century with the pioneering works of Thomas Hodgkin and Rudolph Ludwig Carl Virchow, and since the second half of the 19th century, many iterations of lymphoma classifications have been proposed. During the 1980s, the International Lymphoma Study Group (ILSG) proposed new a classification known as the REAL (Revised European American Lymphoma) classification which was based on identifying lymphoma entities, sorting according to B- and T-cell origin, and defining precursor and mature morphology. In the REAL classification, only one subtype of large B-cell lymphoma was recognised, termed diffuse large B-cell lymphoma, which encompassed a biologically and clinically heterogenous group of lymphomas with shared morphological features. The highly variable architecture, histology, and cytology of DLBCL is a reflection that this lymphoma consists of many different and yet incompletely distinguishable entities (Table 1) [3,53]. In 1995, a new executive committee was organised under the supervision of WHO to achieve a common classification consensus according to morphological, immunophenotypic, and genotypic lymphoma features that had clinical relevance [3,53,54,55,56]. Currently classified hematologic entities, including provisional ones, are described in the WHO “Blue books” (2001, 2008, 2016, 2022; Table 1) and based on a complex of lymphoma features—architectural growth pattern and the origin of neoplastic lymphoid cells defined by morphological, immunophenotypic, molecular genetics, and viral status [3,53,54,55,56,57,58,59,60,61,62,63,64,65,66].

Over the course of subsequent WHO classifications, the number of large B-cell lymphoma entities have proliferated, and some are strongly associated with EBV. Prior to the 2008 WHO classification, a subset of DLBCL, NOS were regarded as EBV+ [67,68]. However, it was not until the 2008 classification that a separate entity was recognised, termed EBV+ DLBCL of the elderly. The rationale was that EBV+ DLBCL was thought to predominantly arise in those over 50 years old, and the presumed aetiology was that of immunoscenecence. In an ageing population, defects in EBV-specific T-cell surveillance were believed to enable an outgrowth of the EBV-infected B-cells [13]. It was later recognised that EBV+ DLBCL also occurred in younger patients [69], and the age criteria was abandoned and the provisional entity was renamed EBV+ DLBCL, NOS [70,71,72]. In fact, studies have reported no significant difference in the incidence of EBV+ DLBCL between younger (<50 years old) and elderly (≥50 years old) patients [52,69].

Both the WHO-HAEM5 and ICC 2022 are unified in their approach to EBV+ DLBCL, which is now regarded as an established entity occurring in patients without immune dysfunction or dysregulation, except for immunoscenecence. EBV+ lymphomas have now been excluded from the DLBCL, NOS category. To render a diagnosis of EBV+ DLBCL, the ICC 2022 classification requires that 80% of the lesional cells are positive, whereas the WHO simply requires that most cells should be EBV+.

### 3.2. Other EBV-Associated Large B-Cell Malignancies

Other large B-cell lymphomas (LBCLs) and lymphoproliferations are consistently associated with EBV, including lymphomatoid granulomatosis, DLBCL, associated with chronic inflammation and fibrin-associated LBCL. Others show EBV infection in a subset of cases such as plasmablastic lymphoma and fluid overload-associated LBCL. Similarly, LBCLs arising in the context of immune deficiency/dysfunction show a more frequent but variable association with EBV.

DLBCL associated with chronic inflammation (DLBCL-CI) occurs in the setting of confined or acquired tissue spaces affected by longstanding inflammation. A typical example would be pyothorax-associated lymphoma in the pleural cavity of patients with longstanding pyothorax. These cases are always associated with EBV and show a clinically aggressive course with a 5-year overall survival of 20–35%. Fibrin-associated large B-cell lymphoma (FA-LBCL) was previously categorised as DLBCL-CI, and comprises a large B-cell proliferation at sites of chronic fibrin deposition. As with DLBCL-CI, FA-LBCL is always associated with EBV, but shows an excellent prognosis with no recorded cases of disseminated disease [73,74].

Lymphomatoid granulomatosis (LG) is another EBV-associated large B-cell lymphoproliferation that occurs in immunocompetent patients and is characterised by angiocentric and angiodestructive lesions in extranodal sites. The lesions comprise large atypical lesional EBV+ B-cells admixed with a prominent reactive T-cell infiltrate. The prognosis of LG depends on the grade; low-grade lesions show a good prognosis and are treated with immune modulation, whereas high-grade lesions are considered to be on a spectrum with EBV+ DLBCL and often require chemotherapy [73].

Some other LBCLs are associated with EBV in most cases. These include primary effusion lymphoma (PEL) and plasmablastic lymphoma (PBL). PEL presents as an effusion in the pleural, pericardial, or peritoneal space in the absence of lymph node involvement or extranodal mass. PEL always shows HHV8 infection and is EBV+ in around 80% of cases, and most cases are associated with HIV infection. The prognosis of PEL is poor, but EBV positivity is associated with a better prognosis. PBL is associated with EBV in approximately 60% of cases and comprises atypical large B-cells with plasmablastic or immunoblastic differentiation. PBL typically affects extranodal sites and shows a dismal prognosis, with an overall survival of 6–32 months [73].

A number of other LBCLs are infrequently or rarely show EBV infection such as HHV8+ DLBCL, high grade B-cell lymphoma/DLBCL with MYC and BCL2/BCL6 rearrangements and primary mediastinal LBCL. The ICC 2022 and WHO-HAEM5 show divergence in their approach to ‘Fluid overload-associated LBCL’ and ‘HHV8 and EBV-negative PEL’, whereby as the name implies, EBV positivity is only permitted in the WHO-HAEM5 classification of this entity [74].

### 3.3. Prevalence of EBV+ DLBCL

Clonal EBV is present in approximately 5% of DLBCL cases in Western countries [10,75,76] and in 5–15% of DLBCL arising in Asian and South-American countries [69,71,77]. However, it should be noted that there is considerable variation in the cut-off applied to define EBV positivity between different studies, where EBER cut-offs between 10–80% have been reported (reviewed in [78] and [79]). Moreover, a recent study has identified rare tumour cells in EBV-negative DLBCL that express EBNA1 detected by RNAscope, but which were negative by conventional EBER staining [80]. These included cases of clonally-related relapsed EBER-negative DLBCL occurring after an initial EBER-positive DLBCL [80]. Thus, these findings support the possibility that loss of the viral genome can occur during disease progression and that this ‘hit and run’ mechanism may be more prevalent than first thought [80].

### 3.4. Viral Gene Expression in EBV+ DLBCL

As described earlier, the EBV-infected B-cells of asymptomatic carriers can express different forms of EBV latency and can also undergo lytic cycle, reflecting the requirement for different gene functions at distinct stages of EBV’s life cycle. EBV+ DLBCL usually expresses a latency II or latency III pattern of virus gene expression (Figure 3) [67,81,82] which could, in part, reflect the origin of EBV+ DLBCL from a different stage of B-cell differentiation. Alternatively, latency II, in which the immunodominant EBNA3 gene family is not expressed, could represent the requirement to minimise the recognition of tumour cells by the immune system in immunocompetent individuals while still retaining critical virally-mediated oncogenic activities such as NF-κB signalling promoted by EBV’s oncogene, LMP1 [83,84]. It is also likely that immune evasion in EBV+ DLBCL might be mediated through the recruitment of an immune suppressive tumour microenvironment (TME) (see Section 3.5).

It has become apparent that mutations in the EBV genome can promote malignancy. One of the best examples of this is the minority of EBV-associated Burkitt lymphoma (BL) that have an unusual form of virus latency in which EBNA2 is deleted [85,86]. These unusual cases are known as ‘Wp-restricted’ BL because expression of the EBNAs is initiated from the Wp promoter as opposed to the Qp promoter that drives EBNA1 expression in conventional BL. Wp-restricted BL cell lines were more resistant to apoptosis than Qp-restricted BL lines, [87,88]. Since then, several studies have shown that genetic variation in the EBV genome also contributes to the pathogenesis of EBV+ DLBCL. Recently, the Wp-restricted genome, P3HR1, was used to infect cord blood humanised (CBH) mice. It was found that although the EBNA2-deleted EBV strain was not capable of transforming cells in vitro [89], in vivo, a subset of mice developed tumours with either a Hodgkin Lymphoma-like or DLBCL-like phenotype [90]. In addition to the lack of EBNA2 expression, these DLBCL-like tumours were found to have low LMP1 expression [90]. This was of particular interest, as in vitro, both EBNA2 and LMP1 are required for B-cell transformation [89,91].

The Kenney lab have also recently shown that B-cells infected with LMP1-deleted EBV can form tumours in vivo in CBH mice [92]. In these mouse models, CD4+ T-cells in the tumour microenvironment were necessary for tumour formation. The CD4+ T-cells provided crucial CD40-ligand signalling in the absence of LMP1, a constitutively active CD40 homolog [92]. Although LMP1 was not necessary for transformation, they also report that LMP1 and LMP2A may cooperate in vivo to promote early-onset lymphomagenesis [93]. In double LMP1 and LMP2A knockouts, fewer tumours were formed in CBH mice, and the tumours that did form grew much slower. In contrast, while LMP2A-deficient tumours were also found to grow more slowly than those expressing LMP2A, the lack of LMP2A did not impact the number of tumours formed [93].

Furthermore, White et al. (2012) showed that B-cells infected with a recombinant virus lacking EBNA3B were more tumorigenic than wild-type (WT) EBV in mice reconstituted with a human immune system [94]. EBNA3B-deficient tumours induced the expansion of EBV-specific T-cells in humanised mice, but these cells failed to infiltrate the tumours. Importantly, truncating mutations in EBNA3B were detectable in tissue samples of some patients with EBV-associated lymphomas, including DLBCL. Taken together, these data suggest that EBNA3B has tumour suppressor functions mediated through its ability to recruit EBV-specific T-cells.

In contrast, in CBH mice, EBNA3C-deleted EBV resulted in fewer DLBCL-like tumours, and those tumours that did form showed 1) reduced tumour growth; 2) increased levels of the tumour suppressor, p16, and type 1 interferon; and 3) higher levels of T-cell infiltration compared with WT EBV [94]. Therefore, although EBNA3C is not always essential for lymphomagenesis, it may play an important role in reducing cellular and immune-mediated tumour suppression.

EBNA3A is known to collaborate with EBNA3C to repress p16 in LCLs [95] and reduce the expression of the pro-apoptotic protein, BIM, in BL cell lines [88,95]. Additionally, BH3-profiling revealed that EBNA3A can upregulate the transcription of the anti-apoptotic protein, BFL1, and improve the localisation of another anti-apoptotic protein, MCL1, to the mitochondria in vitro [96]. In CBH mice, DLBCL-like tumours induced by mutant EBV lacking EBN3A resembled tumours arising from WT EBV infection with similar levels of p16 expression [97]. However, similar to the EBNA3C-deficient tumours, the ENBA3A knockout tumours grow more slowly and with an increased infiltration of CD4+ and CD8+ T-cells [97].

In a recent study, deletions in another part of the EBV genome were observed in DLBCL and other EBV-associated cancers [98]. These deletions result in the loss of genes that are required for completion of EBV’s lytic cycle, e.g., BALF5 [98], while at the same time upregulating the immediate early genes, BZLF1 and BRLF1 [99]. Mutated EBV lacking this region was shown to enhance lymphoma formation by inducing BZLF1 expression in xenograft models [100]. Deletions within the EBV Cp promoter were also reported in EBV+ DLBCL [101]. The Cp promoter usually transcribes the EBNA genes in latency III. Its loss resulted in enhanced in vitro transformation and increased the rate of the progression of EBV+ lymphoproliferative lesions in animal models.

### 3.5. Cellular Genetics of EBV+ DLBCL

Gene expression profiling (GEP) has shown that DLBCL consists of two major subtypes: germinal centre B-cell-like (GCB) DLBCL and activated B-cell like (ABC) DLBCL [63,64]. The GCB and ABC subtypes express genes that are characteristic of normal germinal centre B-cell differentiation or activation of peripheral B-cells, respectively [63,65]. This so-called Cell of Origin (COO) classification of DLBCL can be performed on formalin-fixed paraffin embedded samples (FFPE) using the Hans classifier, but is not as accurate as GEP [42]. In the context of EBV+ DLBCL, generally, studies report a higher prevalence of EBV infection in the ABC-subtype [7], but recent studies in Western countries reported that up to 40% of EBV+ DLBCL were of the GCB subtype [59,66].

Despite the advantages of the COO classification for explaining some of the heterogeneity in DLBCL, the methodology does not fully recapitulate the inter-tumour variability in terms of predicting treatment responses. Instead, genomic studies have revealed that DLBCL consists of many genetic subtypes, with distinct genomic profiles. A recently developed algorithm, LymphGen classifier, identified seven distinct genetic subtypes, namely MCD, N1, A53, BN2, ST2, EZB MYC^+^, and EZB MYC^-^ [102]. In addition to differing genetic signatures, each profile revealed distinct immune microenvironments and treatment outcomes. Additionally, the profiles could be applied to nearly 60% tumours of various DLBCL subtypes, including nodal and extranodal tumours [102]. However, when this algorithm was applied to EBV+ DLBCL, over 80% of tumours could not be attributed to a genetic subtype [103]. This provides further evidence of the unique genetic profile of EBV+ DLBCL compared to its EBV-negative counterpart [103].

Furthermore, GEP in EBV+ DLBCL has demonstrated upregulation of NF-κB signalling and pathways involved in proliferation, cell cycle progression, and metabolism compared to EBV-negative tumours [104]. However, since EBV genes likely contribute to the development of DLBCL, EBV+ tumours appear to have reduced dependency on cellular genetic events. Indeed, genomic profiling has revealed EBV+ tumours have a low mutational burden [32,105] and that mutations in MYD88-mediated TLR-signalling and B-cell receptor signalling pathways are less frequent in EBV+ DLBCL [106], unlike conventional ABC-like DLBCL [107,108]. The exome sequencing of tumours from a cohort of eleven Chinese patients with EBV+ DLBCL (without matched germline DNA) revealed a heterogeneous landscape dominated by mutations associated with a failure of DNA double-strand break–repair by homologous recombination [109]. Recently, targeted sequencing of nine patients identified an elevated frequency of MYC and RHOA mutations together with other genetic aberrations, including mutations in MEF2B and MYD88 [110]. Although the sample size was small, they found that RHOA mutations were predictive of a favourable outcome [110], potentially due to tumour suppressor functions for RHOA; although, this remains to be determined [111]. Kataoka et al. (2019) described a significant enrichment of mutations in TET2 and DNMT3A in EBV+ DLBCL [112]. Gebauer et al. (2021) used whole-genome and targeted sequencing with FISH to analyse 47 EBV+ DLBCL. They found that EBV+ DLBCL was genetically distinct from EBV-negative DLBCL due to frequent mutations in ARID1A, KMT2A/KMT2D, ANKRD11, and NOTCH2 [105]. Gene set enrichment analysis identified that mutated genes were enriched in NFκB, IL6/JAK/STAT, and WNT signalling pathways [105]. They also found large deletions on chromosome 6 to be a highly recurrent feature of EBV+ DLBCL [105].

DLBCL/high-grade B-cell lymphoma with *MYC* and *BCL2* and/or *BCL6* rearrangements carries a particularly poor prognosis [113,114]. Conventionally, these tumours are known as double or triple hit lymphomas (DHL/THL). EBV is only rarely found in association with DHL/THL; a recent study examining a cohort of 846 DHL/THL found that 16 cases were EBV+, 13 of which were of the GCB type [115]. Another study by Frontzek et al.l (2023) found that 4% of tumours carried translocations of *MYC* and *BCL6*, but no cases of *MYC/BCL2* double hit [103]. Nonetheless, whether synergistic effects between EBV and *MYC/BCL2/BCL6* aberrations contribute to the pathogenesis of this subset of tumours remains to be established.

### 3.6. The Tumour Microenvironment of EBV+ DLBCL

The development of EBV+ DLBCL in some individuals with apparently healthy immune systems argues against the requirement for a systemic loss of EBV control in all cases, particularly in younger, immunocompetent patients [116]. To explain this, focus has begun to shift towards an exploration of the tumour microenvironment (TME) as a contributing factor. Thus, EBV+ DLBCL displays a tolerogenic TME with an increased expression of PDL1, PDL2, LAG3, and TIM3 immune checkpoints [82,117,118]; raised levels of immunosuppressive cytokines (e.g., IL10) [118]; and higher pro-tumoral loads of CD163/CD68 “M2” macrophages [117]. A recent study by Carreras et al. (2022) further found that pentraxin 3 (PTX3), a marker of the M2c-like macrophage subtype and NF-κB activation, correlated with a poorer prognosis. These CD163 and PTX3 co-expressing M2-like macrophages were also found at higher levels in EBV+ DLBCL than EBV-negative tumours [119]. This regulatory environment co-exists with increased numbers of CD8+ T-cells and granzyme B+ cytotoxic effector cells, known as an ‘inflamed phenotype’, interpreted as an ineffective host immune response to virus-infected tumour cells [81,117,118]. The potential importance of targeting this tolerogenic TME in EBV+ DLBCL is evidenced in a study by Ma et al. (2016) which used PD-1 and CTLA4 inhibition to increase the EBV-specific T-cell response in humanised mice implanted with EBV+ cord blood cells [120]. This use of immune checkpoint blockade facilitated the T-cell-mediated control of tumour out-growth [120].

Although previous studies provide some insights into the nature of the TME of EBV+ DLBCL, they have mainly used bulk RNA-seq or low-plex immunohistochemistry. Consequently, we still lack a detailed spatial characterisation of the TME of EBV+ DLBCL and an understanding of how it varies compared to other DLBCL subtypes. Moreover, although LMP1 has been reported to increase the expression of some immunosuppressive molecules in vitro (e.g., PDL1, IL10) [83], we also lack deeper mechanistic insights into the contribution of different oncogenic drivers, including EBV, to the DLBCL TME.

## 4. Conclusions and Perspectives

EBV was first identified as a potent transformer of B-cells and was causally associated with the development of BL. Since then, EBV has been shown to be associated with a diverse range of different cancer types, including DLBCL. The pathogenesis of EBV+ DLBCL remains poorly understood and several key questions remain to be answered. Unravelling the complex interplay between the different aetiological factors and EBV infection, and how this contributes to transformation, will not only reveal new insights into the pathogenesis of DLBCL, but is also likely to lead to the development of novel therapies for treating this aggressive malignancy.

## Figures and Tables

**Table 1:** Modifications to aggressive B-cell lymphoma classification in 2022 WHO and ICC classification, and the frequency of EBV involvement.

WHO Classification 2017	WHO Classification 2022	ICC Classification 2022	EBV Status
1. Diffuse large B-cell lymphoma NOS	1. Large B-cell lymphomas		
DLBCL, NOS	DLBCL	DLBCL	Negative by definition
2. Other lymphomas of large B-cells			
T-cell/histiocyte-rich LBCL	T-cell/histiocyte-rich LBCL	T-cell/histiocyte-rich LBCL	Negative
Primary DLBCL of the CNS	Primary large B-cell lymphoma of immune-privileged sites (CNS, vitreoretinal, testicular)	Primary DLBCL of immune-CNS, Primary DLBCL of testis *	Predominantly negative
Primary cutaneous DLBCL, leg type	Primary cutaneous DLBCL, leg type	Primary cutaneous DLBCL, leg type	Negative
EBV-positive DLBCL, NOS	EBV-positive DLBCL	EBV-positive DLBCL, NOS	100% positive by definition
Primary mediastinal (thymic) LBCL	Primary mediastinal LBCL	Primary mediastinal LBCL	Rare examples of EBV-positive cases reported
Intravascular LBCL	Intravascular LBCL	Intravascular LBCL	Negative
DLBCL associated with chronic inflammation	DLBCL associated with chronic inflammation	DLBCL associated with chronic inflammation	100% positive
Lymphomatoid granulomatosis	Lymphomatoid granulomatosis	Lymphomatoid granulomatosis	100% positive
ALK-positive LBCL	ALK-positive LBCL	ALK-positive LBCL	Negative
Plasmablastic lymphoma	Plasmablastic lymphoma	Plasmablastic lymphoma	70–80% positive
LBCL with IRF4 rearrangement	LBCL with IRF4 rearrangement	LBCL with IRF4 rearrangement *	Negative
Burkitt-like lymphoma with 11q aberration	High-grade B-cell lymphoma with 11q aberrations	LBCL with 11q aberrations	Negative
(Previously included in DLBCL associated with chronic inflammation)	Fibrin-associated LBCL	Fibrin-associated DLBCL	~100% positive
(Not previously included)	Fluid overload-associated LBCL	HHV8 and EBV-negative PEL	WHO:13–30% positive. ICC: negative by definition.
	2. KSHV/HHV8-associated B-cell LPD		
HHV8-positive DLBCL, NOS	KSHV/HHV8-positive DLBCL	HHV8+ DLBCL	Occasional cases are positive
Primary effusion lymphoma	Primary effusion lymphoma	Primary effusion lymphoma	Most cases positive
3. B-cell lymphoma unclassifiable	1. Large B-cell Lymphoma (continued)		
B-cell lymphoma, unclassifiable, with features intermediate between DLBCL and classical Hodgkin lymphoma	Mediastinal grey zone lymphoma	Mediastinal grey zone lymphoma	Negative
4. High-grade B-cell lymphoma			
High-grade B-cell lymphoma with *MYC* and *BCL2* and/or *BCL6* rearrangements	DLBCL/high grade B-cell lymphoma with *MYC* and *BCL2* rearrangements	High grade B-cell lymphoma; *MYC:BCL2* or *MYC:BCL6* or *MYC:BCL2:BCL6*	Negative (rare reported EBV-positive cases)
High-grade B-cell lymphoma, NOS	High-grade B-cell lymphoma, NOS	High-grade B-cell lymphoma, NOS	Negative

* denotes provisional entity.

## Data Availability

Not applicable.
